# Daily Effect of Recovery on Exhaustion: A Cross-Level Interaction Effect of Workaholism

**DOI:** 10.3390/ijerph15091920

**Published:** 2018-09-04

**Authors:** Monica Molino, Claudio G. Cortese, Chiara Ghislieri

**Affiliations:** Psychology Department, University of Turin, Via Verdi 10, 10124 Turin, Italy; monica.molino@unito.it (M.M.); chiara.ghislieri@unito.it (C.G.)

**Keywords:** workaholism, recovery, exhaustion, work-related diseases, diary study

## Abstract

Workaholics generally allocate an excessive amount of time and energy to their work at the expense of having time for recovery from work. Nevertheless, a complete recovery is an essential prerequisite for well-being. This study examines the moderating role of workaholism in the relationship between daily recovery and daily exhaustion. Data were collected among 95 participants who completed a general questionnaire and a diary booklet for five consecutive working days. Multilevel analysis results confirmed a cross-level interaction effect of workaholism, showing that the negative relationship between recovery and exhaustion at the daily level is weaker for those with a high (versus low) level of workaholism. These insights suggest the promotion of interventions aimed at addressing workaholism among workers, and the design of projects able to stimulate recovery from work, particularly for workaholics.

## 1. Introduction

Due to the increasing of work intensity and job uncertainty in recent years, many employees have to face a high work pace, fixed deadlines, and insufficient time to do their tasks [1,2], with the consequent need to work hard, also in the evenings and weekends. However, some of them work in an excessive way not only for external requirements or for economical necessities, but because they feel they have to [3]. This over-commitment of energy and time to work has been used in the literature to describe the notion of workaholism [4].

Workaholism is considered as one of the most common current addictions in many Westernized countries. These cultures value success and accomplishment, and consider work as a central life aspect, essential to be socially accepted, to feel integrated and to obtain freedom and personal independence. In these societies, workaholics’ characteristics are generally appreciated, to the extent that, according to some authors, the society itself facilitates and enhances workaholism tendencies [5].

So far, there has been a lack in the number of reliable prevalence estimates of workaholism, also in Italy. A recent nationally representative study among 1124 employees in Norway revealed that 8.3% of them were workaholics [6]. Sussman, Lisha and Griffiths [7] estimated a prevalence of workaholism as 10% of the U.S. adult population. Based on the workaholism literature as a whole, the prevalence of workaholism in the general population has been estimated ranging from 5% to 10% [7], and even up to 25% [8].

Today’s organizations increasingly push their employees to work harder and longer to remain successful in the global competition [9,10,11,12], to the point of considering workaholism convenient and rewarding it. Furthermore, with the expansion of technology, individuals can always stay connected to their work [13]. Taken together, these changes lead employees to work harder than before [3] and for some of them work becomes an escape route, useful to hide difficulties in living their lives and relationships [5].

Working hard does not represent a problem as long as workers can recover from the work-related efforts on a daily basis [14], since sufficient recovery is an essential prerequisite for well-being [15]. Nevertheless, workaholics are so involved in their work that they find it very difficult to detach from it; thus, they seem to neglect their need for recovery [16]. Workaholics spend much time and energy at work, leaving fewer resources to devote to their family and to their activities of non-work life [17]. Consequently, they have relatively few opportunities to recover from their workdays and they generally suffer from work–family conflict and exhaustion [16].

The aim of the present study was to examine the relationship between recovery and exhaustion at the daily level, and the cross-level interaction effect of workaholism. The study used a within-person daily diary approach that covered five consecutive working days. The vast majority of studies on workaholism and recovery have investigated between-person differences throughout cross-sectional research, while few studies have used the daily approach so far [2,18]. While the cross-sectional method considers the person as the unit of analysis based on interindividual variation, diary designs permit one to analyse short-term intraindividual fluctuations on a daily basis. For this reason, such a method has been indicated as the most appropriate in work and organizational research, particularly in the areas of health and stress, work–home interface, and recovery [19].

### 1.1. Workaholism

The term workaholism was first used by Oates in 1971 [20] to describe an excessive and uncontrollable need to work that permanently disturbs health, happiness and relationships. More recently, Andreassen and colleagues [21] defined workaholism as “being overly concerned about work, being driven by an uncontrollable work motivation, and spending so much energy and effort on work that it impairs private relationships, spare-time activities and/or health” [21] (p. 265).

Currently, there is not an agreed-upon definition of workaholism [4]. Nevertheless, scientific interest in this topic is growing since it is considered able to impact different areas of human functioning, at the individual, family, organizational and societal levels [22]. Despite work addiction not generally being accepted as a clinical condition and not being officially listed in the Diagnostic and Statistical Manual (DSM-5) [23], many writers have conceptualized it as pathology.

Following the component model of addiction [24], workaholism can be defined according to seven general criteria [25]: cognitive and/or behavioural salience (preoccupation with work); mood modification (working in order to escape or avoid dysphoria); reduced tolerance (working increasingly more to achieve the same mental and physiological effect); withdrawal (dysphoria when prohibited from working); conflict (work comes in conflict with one’s own and others’ needs and lives); relapse (falling back into old patterns after a period of improvement); and health problems (working so much that health, relationships and other aspects of one’s own life are negatively affected). Building on this model, Andreassen and colleagues [21] developed the Bergen Work Addiction Scale (BWAS) for the assessment of workaholism.

Although some researchers highlighted some positive aspects of workaholism, such as high work motivation [26,27] or work passion [28,29], today the prevailing perspective suggests that workaholism comprises negative consequences on different areas of human functioning [4,6,22].

Regarding consequences for individuals, several studies have found a positive relationship between workaholism and burnout, a state of exhaustion and depletion of mental resources [16,30,31,32]. Since workaholics spend excessive amounts of energy and effort at work, they might exhaust their energy back-up and burn out [18,33]. Among the other health outcomes, we can find anxiety/insomnia, somatic symptoms and social dysfunction [30]. Moreover, workaholism adversely affects also the non-work domain, increasing work–family conflict [16,17,34]. Indeed, workaholics spend a lot of time and energy on their work, also in the evening, during weekends and holidays, at the cost of their family life and relationships. For families, it is difficult to recognise and deal with a member’s workaholic behaviours and, generally, families need help as much as the workaholic does [10].

Furthermore, workaholism can negatively impact the workplace. Research demonstrated that high levels of workaholism are related to greater aggressive behaviours at work [35]; moreover, workaholics are not able to work in a team, delegate to others, communicate and show their emotions [36]. Organizations seem to aim for short-term results of workaholism, neglecting its possible detrimental long-term effects, in terms of reduced job and life satisfaction, impaired health and well-being, and, consequently, decreased efficiency and productivity.

In short, some authors indicated workaholics as a risk group for ill-health [37]; in this study, a positive relationship between workaholism and exhaustion was expected.

**Hypothesis** **1.** *General workaholism is positively associated with day-level exhaustion*.

### 1.2. Recovery

Recovery refers to the process during which an individual’s functioning that has been called upon during a stressful experience returns to its prestressor levels, reducing strain [38]. When recovery is not sufficient, individuals have to put in extra effort at work to maintain a satisfactory performance level, which may inflict strain and in the long period lead to health problems [38,39].

Sonnentag and Fritz [40] referred to the Effort-Recovery model [38] and to the Conservation of Resources theory [41] to develop an understanding of recovery experiences. According to the Effort-Recovery model, effort expenditure at work leads to load reactions such as fatigue or physiological activation. Under optimal circumstances, which mean that the individual is no longer exposed to the work demands, load reactions are released and recovery occurs. Consistent with this model, a precondition for recovery is that the functional systems strained during work will not be called upon any longer. However, if no adequate recovery takes place, stress-related load reactions do not return to prestressor levels [42]. Moreover, continuous exposure to high work demands and incomplete recovery could induce an accumulation of load reactions leading to chronic health impairment [2,42]. The Conservation of Resources theory [41] assumes that stress occurs when an individual’s resources are threatened or lost. Therefore, to recover from stress, individuals have to restore their resources and gain new resources. Stress recovery on a day-to-day basis particularly refers to internal resources such as energy or positive mood.

According to these two theories, refraining from work demands and avoiding activities in the extra-work time that call upon the same functional systems, or internal resources, as those required at work, are important strategies. Moreover, gaining new internal resources such as energy or positive mood will additionally help to restore endangered resources [40].

According to the Sonnentag and Fritz model [40], recovery can be achieved through some off-job activities that permit one to replenish used personal resources and/or to build new ones. Four varieties of activities have been identified: psychological detachment, relaxation, mastery and control [40]. Psychological detachment implies refraining from job-related activities and mentally disengaging from work during off-job time. The term detachment has been used to describe an individual’s sense of being away from the work situation [43]. It means that individuals need to stop thinking about work and not to be occupied by work-related affairs and problems when they are not working. Relaxation refers to feelings of peacefulness and calm, low activation and increased positive affect, and is generally associated with leisure activities. Mastery refers to being occupied in off-job activities that distract from one’s own job and provide the opportunity to learn new skills and develop new personal resources, such as self-efficacy and positive mood, in other domains. Finally, control represents the degree to which a person may decide which activity to pursue in his/her leisure time, when and how, and may increase positive reactions, self-efficacy and feelings of competence.

Empirical evidence suggests that recovery is helpful in restoring from job strain and is negatively related to health complaints, depressive symptoms, exhaustion and sleep problems [40,44,45,46,47,48,49]. Therefore, this study hypothesized a negative relationship between recovery during evening hours and exhaustion at bedtime.

**Hypothesis** **2.** *Day-level recovery is negatively related to day-level exhaustion*.

The workaholics’ tendency to devote more resources to their work than to their non-work life leads them to neglect their activities outside the job. Addictive workers are unable to reduce or control hard work, they continue to work despite social or health problems, and they experience unpleasant withdrawal symptoms when away from work [18,27]. These behaviours reduce workaholics’ resources when they are not working, in terms of time, energy, psychological and mental resources that could be invested in non-work activities.

The prolonged exposure to work demands and thoughts during off-job hours does not allow workaholics to complete recovery before the next working day starts [2,18]. Therefore, we expected a cross-level interaction effect of workaholism on the negative relationship between recovery and exhaustion at the daily level.

**Hypothesis** **3.** *General workaholism negatively moderates the relationship between day-level recovery and day-level exhaustion, so that day-level recovery has a weaker negative relationship with day-level exhaustion for individuals with a high (versus low) level of workaholism. In other words, for workaholics, the recovery’s ability to reduce exhaustion is weaker than for non-workaholics*.

**Figure 1:**
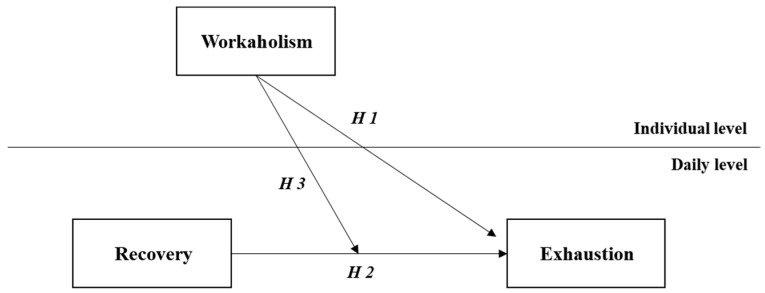
Hypothesized model.

Figure 1 depicts study hypotheses.

## 2. Materials and Methods

### 2.1. Participants and Procedure

The study involved a heterogeneous sample of 95 Italian workers, which included 58 females (61%) and 37 males (39%). Their mean age was 36.24 (*SD* = 10.41); 72% were married or cohabited and 64% did not have children. In the sample, 42% had a bachelor’s or master’s degree or a higher educational qualification, 39% finished high school and the others had a lower level of education.

Among participants, 79% were employees, 14% were self-employed workers and the remaining participants had different types of contracts; 25% were working in the industrial sector, 19% in commerce, 11% in the education and research sector, 11% in private service, 10% in public services and 24% indicated another sector. The mean working hours per week were 40.02 (*SD* = 8.40). Mean seniority on the job was 10.31 years (*SD* = 10.08).

We used a snowball sampling procedure to recruit participants. We started from a convenience sample of 40 workers, randomly identified by researchers, and asked them to provide the contact information of their friends or family members with a job, in order to enlarge the sample. Participants who agreed to be involved received a diary booklet. They were instructed to fill in a general questionnaire, in which they provided some demographic data and information on the general level of the measured variables, before starting with the diary. Then they were asked to fill in the diary for five consecutive working days at the end of each day before going to bed. A total of 95 usable diaries were returned.

Participation in the study was voluntary, anonymous and no compensation was offered. The research was conducted in line with the Helsinki Declaration [50], as well as the data protection regulation of Italy (Legislative Decree No. 196/2003). A cover letter attached to the questionnaire provided information about the study aims, anonymity and data treatment, and instructions for filling out the questionnaire. Agreeing to fill out the questionnaire, all study participants provided their informed consent.

### 2.2. Measures

*General Workaholism* was measured with the 7-item Bergen Work Addiction Scale (BWAS) [21] in its Italian version [51]. A sample item is “How often during last year have you thought of how you could free up more time to work?” (from 1 = *never* to 5 = *always*); Cronbach’s alpha for this study was 0.81.

### 2.3. Diary Booklet

The diary booklet consisted of five identical questionnaires, one for each day, from Monday to Friday. Participants responded to all day-level measures on a seven-point scale (from 1 = *strongly disagree* to 7 = *strongly agree*). They were asked to fill-in the diary every evening before going to bed and to answer thinking about the day and not about their overall situation.

*Day-level Exhaustion* was assessed using the 8 items taken by the Oldenburg Burnout Inventory (OLBI) [52]. A sample item is “Today, I felt emotionally drained by my work”. Cronbach’s alpha was calculated separately for each day and ranged from 0.68 to 0.87 (*M* = 0.75) in this study.

*Day-level Recovery* was measured by the Recovery Experience Questionnaire [40] in its 12-item Italian version [53]. An example item is: “This evening, after work, I forgot about work”. Cronbach’s alpha for this study ranged from 0.94 to 0.96 (*M* = 0.95).

### 2.4. Statistical Analysis

Data collection led to a two-level model with 475 occasions at the within/daily level (Level 1) and 95 participants at the between/individual level (Level 2). Given the nested structure of our data, we tested multilevel structural equation modeling (ML-SEM) using the statistical software Mplus 7 (Muthén & Muthén, Los Angeles, CA, USA) [54]. The method of estimation was robust maximum likelihood (MLR). Daily recovery was considered as predictor at Level 1 and general workaholism as predictor at Level 2. 

We first ran an intercept-only model (Model 0) that indicated the amount of variance in the dependent variable (i.e., exhaustion) which was explained by differences between individuals. For Model 0 we calculated the Intraclass Correlation Coefficient (ICC), which represents the proportion of variance that lies between individuals. The ICC for exhaustion was 0.58, indicating a moderately high clustering effect and justifying the multilevel approach [55]. We then proceeded to test a series of two-level ML-SEMs predicting exhaustion at Level 1.

In Model 1 (random intercept model), we added Level 1 predictor (i.e., recovery) as fixed effect, specifying the intercept term at Level 1 as a random variable. In Model 2 (random slope model), we tested Level 1 predictor (i.e., recovery) as random effect. In Model 3 (intercept as outcome model), we added the between-level variable (i.e., workaholism) as predictor of the intercept. Finally, in Model 4 (intercept and slope as outcomes model), we examined the cross-level interaction effect, testing whether the Level 2 predictor (i.e., workaholism) predicted the between variability of both intercept and slope. All predictors were grand-mean centered to facilitate the interpretation of main and conditional effects [56]. In order to test nested models we evaluated the difference between the deviances through the likelihood ratio (LR) test.

The statistical software SPSS Statistics 24 (IBM, Armonk, NY, USA) was used to test descriptive data analysis, Pearson correlations and Cronbach’s alpha.

## 3. Results

### 3.1. Descriptive Statistics

Descriptive statistics and correlations between study variables are presented in Table 1. All significant relationships between variables were in the expected direction. Day-level recovery negatively correlated with day-level exhaustion. Moreover, general workaholism showed a negative correlation with day-level recovery and a positive correlation with day-level exhaustion.

### 3.2. ML-SEM Analyses

The results of the analyses of multilevel models are displayed in Table 2. As reported in Model 1, increases in daily recovery deviations at Level 1 predicted lower mean levels of exhaustion, confirming Hypothesis 1. Moreover, Model 3 showed a positive association between general workaholism and daily-exhaustion, confirming Hypothesis 2. Finally, in order to test the cross-level interaction effect, in Model 4 we entered an effect of workaholism at level 2 on the intercept and slope of the relationship between recovery and exhaustion at Level 1 and we found a significant effect. The LR test confirmed significant reduction of unexplained variance at each step of the analysis.

**Figure 2:**
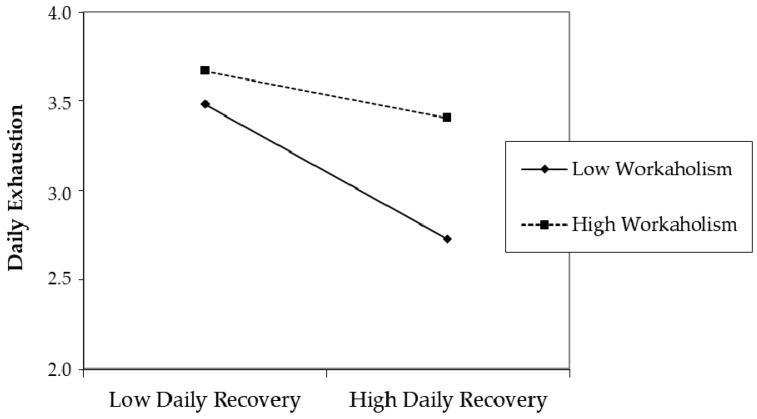
The interaction effect between general workaholism and daily recovery in the prediction of daily exhaustion.

Figure 2 graphically represents the interaction effect, differentiating between low and high levels of workaholism, defined as one standard deviation below and above the mean [57]. As the figure shows, the negative relationship of daily recovery with daily exhaustion was weaker for high (versus low) levels of workaholism. Thus, also Hypothesis 3 was confirmed.

## 4. Discussion

The present diary study aimed at examining, for the first time, whether the negative relationship between recovery and exhaustion, measured at the daily level, differs between individuals with high versus low levels of workaholism. First of all, results supported Hypothesis 1, showing a positive relationship between workaholism and daily exhaustion. Particularly, this finding confirmed that workaholism may have negative effects on individual well-being, as previously demonstrated [30,58,59,60], and calls for a greater attention to this phenomenon and its risks.

Moreover, according to the Effort-Recovery model, adequate recovery from work is negatively related to long-term health impairment [38,40] and some empirical evidence presented in the literature supports this model [45,46,48,49]. Our results also confirmed the recovery’s ability to reduce exhaustion (Hypothesis 2), providing stronger evidence for this relationship since it was investigated at the daily level.

Finally, Hypothesis 3 suggested a moderational role of workaholism and investigated it using a multi-level approach. Results confirmed the cross-level interaction effect and indicated that the recovery’s ability to decrease exhaustion is weaker for those individuals with high (versus low) levels of workaholism. The workaholics’ tendency to devote more resources to their work than to their non-work life leads them to neglect their activities outside the job and, consequently, their efficacy in recovering from work. Workaholics, indeed, tend to work long hours not only for external requirements or needs, but because they often feel they have not done enough yet [2,3], and even when they are not involved in job-related activities after work they continue to think about work and feel guilty. Thus, they are so involved in their job-related thoughts and affairs that it seems particularly difficult for them to effectively recover. As a previous study suggested [18], for workaholics, more so than for non-workaholics, what kind of activities they do during leisure time seems to be important, since not all types of recovery experiences are able to reduce their exhaustion. For example, Bakker and colleagues [18] found that time spent in sport and physical exercise during off-job time was more positively associated with evening well-being for workaholics than for non-workaholics. On the contrary, the kind of recovery experiences considered in our study showed a weaker effect on exhaustion for individuals with high levels of workaholism.

### Limitations

This study has some limitations that should be mentioned. The first one is that data relied on self-report, raising concerns about common method variance [61]. Future research should consider also other ratings (e.g., colleagues, supervisors or partners) and objective ratings, so as to avoid this problem.

Secondly, results mainly concerning relationships between variables considered in this study are correlational in nature [62]. Despite the multilevel approach, inferences about causality are quite limited and could not be made with confidence. Moreover, the study focused on a convenience heterogeneous sample of workers. Therefore, we should be cautious with generalizing the results.

A further limitation of the study is related to the snowball sampling procedure that may have influenced the sample structure, hence not representing the population as a whole. Moreover, participants could have had a special interest in this study that led them to accept to be involved. Nevertheless, mean levels of both general workaholism (*M* = 2.17, *SD* = 0.80) and general exhaustion (*M* = 2.37, *SD* = 0.46) were in line with a previous study [63] that involved an Italian sample of 617 workers and applied the same instruments used in this study to detect workaholism (*M* = 2.23, *SD* = 0.73) and exhaustion (*M* = 2.36, *SD* = 0.55).

Another limitation concerns the recovery construct, since we used a general measure of recovery and could not draw more specific conclusions about the effect of different kinds of leisure activities on workaholics’ exhaustion. Moreover, this study focused specifically on experiences with potential for recovery during off-job time, although research has indicated that recovery may occur during working time as well [64].

Despite its limitation, a strong point of the current study is that it examined this pattern of relationships on a daily basis, respecting the call for more within-person studies on both workaholism and recovery [2].

## 5. Conclusions

First of all, this study highlighted the importance of reaching awareness of the existence of the often ignored or underestimated phenomenon of workaholism. Individuals and organizations must understand the problem, its causes and costs, and consider viable solutions, especially for those jobs characterized by increase of work intensity and job uncertainty and by permeable boundaries between work and the rest of life [65]. In particular, this responsibility lies on managers and supervisors who can more easily than others witness, detect and signal workaholic behaviours, and suggest and monitor alternative ones. Employers and managers play a crucial role, as they can be a model and set a good example to work in a healthy way [12]. Programmes for leadership development [12,66,67] in the work context and attention to employer-recruitment selection represent important interventions. Leaders can also actively help their collaborators to behave less dysfunctionally: encouraging them to delegate some of their work, monitoring their job planning and priorities, giving them specific time to take breaks and to leave work [68].

This study also confirmed previous findings that indicated recovery from work during off-job time as a crucial experience for protecting individuals’ well-being [40]. Despite the fact that it may be difficult for employees to find a good interaction between all their different priorities (their work, their family, their leisure activities), they need to be supported in understanding the importance of engaging in different activities during off-job time, and to mentally detach from work on a daily basis [39,69]. One way to enhance recovery is to keep one’s work life separate, as much as possible, from one’s non-work life [48]. Organizations should encourage and support this segmentation practice addressing the implicit norms of unlimited availability and the use of communication devices (e.g., smartphones, laptops with e-mail access) that make it difficult to mentally and physically detach from work during off-job hours [18]. In general, organizations should question the necessity of the culture of long working hours to support employees finding a healthy work–life balance [39]. Moreover, time management training can be considered an important intervention, especially for employees with tendencies toward working excessively, to gain conscious control over their time schedule by setting realistic goals and prioritizing tasks [2].

## Figures and Tables

**Table 1:** Means, standard deviations and correlations among the study variables.

Variables	*M*	*SD*	1	2	3
1. General workaholism	2.17	0.80	-		
2. Day-level recovery	4.46	1.28	−0.18 **	-	-
3. Day-level exhaustion	3.29	0.98	0.29 **	−0.36 **	-

Note: ** *p* < 0.001. Day-level data was averaged across the 5 days.

**Table 2:** Results of multilevel structural equation modeling predicting day-level exhaustion (unstandardised estimates).

Effects	Model 0			Model 1			Model 2			Model 3			Model 4		
	Est.	SE	CI 95%	Est.	SE	CI 95%	Est.	SE	CI 95%	Est.	SE	CI 95%	Est.	SE	CI 95%
**Fixed effects**															
Intercept (γ_00_)	3.29	0.07	(3.12, 3.41)	3.28	0.08	(3.14, 3.44)	3.32	0.07	(3.20, 3.46)	3.31	0.08	(3.17, 3.45)	3.34	0.08	(3.18, 3.46)
Recovery (γ_10_)				−0.21	0.04	(−0.29, −0.14)	−0.21	0.04	(−0.29, −0.12)	−0.20	0.05	(−0.30, −0.12)	−0.20	0.05	(−0.29, −0.11)
Workaholism (γ_01_)										0.28	0.09	(0.06, 0.41)	0.26	0.09	(0.06, 0.40)
Rec * Wsm (γ_11_)													0.11	0.05	(0.02, 0.22)
**Random effects**															
Level 2															
Intercept (t_00_)	0.55	0.10	(0.40, 0.81)	0.49	0.09	(0.36, 0.72)	0.42	0.09	(0.27, 0.60)	0.39	0.07	(0.30, 0.53)	0.36	0.08	(0.24, 0.54)
Daily recovery slope (t_11_)							0.07	0.03	(0.04, 0.14)	0.07	0.03	(0.04, 0.13)	0.07	0.02	(0.03, 0.12)
Level 1															
(σ^2^)	0.39	0.03	(0.34, 0.46)	0.37	0.03	(0.32, 0.44)	0.34	0.03	(0.28, 0.39)	0.33	0.03	(0.28, 0.38)	0.33	0.02	(0.29, 0.38)
**Deviance**															
(−2*log likelihood)	1105.03			1062.96			1046.11			1036.73			1031.66		
Diff −2*log likelihood				42.07 ***			16.85 ***			9.38 **			5.07 *		

Notes: *** *p* < 0.001; ** *p* < 0.01; * *p* < 0.05; CI = confidence interval. Day-level data was averaged across the 5 days.
